# Analysis of the Significance of Immune Cell Infiltration and Prognosis of Non-Small-Cell Lung Cancer by Bioinformatics

**DOI:** 10.1155/2021/3284186

**Published:** 2021-09-22

**Authors:** Heng Sun, Bowen Sui, Yu Li, Jun Yan, Mingming Cao, Lijia Zhang, Songjiang Liu

**Affiliations:** ^1^The First Affiliated Hospital of Heilongjiang University of Chinese Medicine Department of Oncology, Harbin, China; ^2^The First Affiliated Hospital of Heilongjiang University of Chinese Medicine Department of Oncology Respiratory, Harbin, China; ^3^Heilongjiang University of Chinese Medicine, Harbin, China; ^4^The First Affiliated Hospital of Heilongjiang University of Chinese Medicine Department of Oncology Ethics Office, Harbin, China

## Abstract

**Objective:**

To perform gene set enrichment analysis (GSEA) and analysis of immune cell infiltration on non-small-cell lung cancer (NSCLC) expression profiling microarray data based on bioinformatics, construct TICS scoring model to distinguish prognosis time, screen key genes and cancer-related pathways for NSCLC treatment, explore differential genes in NSCLC patients, predict potential therapeutic targets for NSCLC, and provide new directions for the treatment of NSCLC.

**Methods:**

Transcriptome data of 81 NSCLC patients and the GEO database were used to download matching clinical data (access number: GSE120622). Form the expression of non-small cell lung cancer (NSCLC). TICS values were calculated and grouped according to TICS values, and we used mRNA expression profile data to perform GSEA in non-small-cell lung cancer patients. Biological process (GO) analysis and DAVID and KOBAS were used to undertake pathway enrichment (KEGG) analysis of differential genes. Use protein interaction (PPI) to analyze the database STRING, and construct a PPI network model of target interaction.

**Results:**

We obtained 6 significantly related immune cells including activated B cells through the above analysis (Figure 1(b), *p* < 0.001). Based on the TICS values of significantly correlated immune cells, 41 high-risk and 40 low-risk samples were obtained. TICS values and immune score values were subjected to Pearson correlation coefficient calculation, and TICS and IMS values were found to be significantly correlated (Cor = 0.7952). Based on non-small-cell lung cancer mRNA expression profile data, a substantial change in mRNA was found between both the high TICS group as well as the low TICS group (FDR 0.01, FC > 2). The researchers discovered 730 mRNAs that were considerably upregulated in the high TICS group and 121 mRNAs that were considerably downregulated in the low TICS group. High confidence edges (combined score >0.7) were selected using STRING data; then, 191 mRNAs were matched to the reciprocal edges; finally, an undirected network including 164 points and 777 edges was constructed. Important members of cellular chemokine-mediated signaling pathways, such as CCL19, affect patient survival time.

**Conclusion:**

(1) The longevity of patients with non-small-cell lung cancer was substantially connected with the presence of immature B cells, activated B cells, MDSC, effector memory CD4 T cells, eosinophils, and regulatory T cells. (2) Immune-related genes such as CX3CR1, CXCR4, CXCR5, and CCR7, which are associated with the survival of NSCLC, affect the prognosis of NSCLC patients by regulating the immune process.

## 1. Introduction

The occurrence of tumors is not only related to the biological characteristics of the tumor cells themselves. And the interaction between cancer cells and tumor immune microenvironment and immune system also plays a very significance role. One of the problems that should be investigated is whether immune cell infiltration in tumor tissues is connected to tumor treatment and prognosis.

Lung carcinoma is a major cancer that has a high risk of morbidity and mortality, and data show that one person dies from lung cancer every 30 s worldwide [1] Lung tumor is divided into 2 types: small-cell lung cancer (SCLC) and non-small-cell lung cancer (NSCLC). Small-cell lung cancer (SCLC) accounts for around 85% of all lung cancer cases [2]. Because of the insidious onset, diversity of clinical manifestations, and lack of specificity of primary lung cancer patients, most patients are already in the middle and late stages of the disease when they are diagnosed with lung cancer, so whether lung cancer patients can be screened, diagnosed, and treated early becomes a key factor in prognosis.

The tumor microenvironment (TME) is a bidirectional, dynamic, and intricate network system composed of various cells (T cells, B lymphocytes, macrophages, NK cells, etc.) and extracellular components (cytokines, chemokines, and receptors, etc.), and each element of this network is capable of promoting malignant transformation and influencing both the onset, progression, and metastasis of lung cancer, development and metastasis, and the response to drug therapy [3] T-cell surface programmed cell death protein 1 (PD-1) in TME has been demonstrated to bind to programmed cell death ligand-1 (PD-L1) on the surface of cancer cells in recent years, causing T-cell dysfunction or depletion, resulting in immune tolerance and tumor cell response. Immune tolerance occurs, leading to immune escape of tumor cells [4], and in the process of killing tumor cells, the most central role in TME is played by CD8+ T cells [5], the quality and quantity of which are critical for immunotherapeutic efficacy [6]. Studies of PD-1 inhibitors for melanoma [7] have found that the number of CD8+ T cells correlates with efficacy. Tumor-infiltrating B cells are an important part of the immune system's microenvironment. In NSCLC patients, CD4+ T lymphocytes can be seen in tumor cells infiltrated by B cells and tumor cells with tertiary lymphoid structure. And high density of follicular B cells is associated with a better prognosis in NSCLC patients [8]. Bruno et al. [9] found that tumor-infiltrating B cells can present endogenous tumor antigens to CD4+ TIL and alter them in vitro. It has also been reported that bone marrow-derived suppressor cells (MDSCs) are able to produce factors like TGF-p and IL-10, which promotes the development of initial CD4+ T cells into Treg cells, resulting in the increase of Treg cells, thereby suppressing body immune response [10]. In addition, MDSC can block the secretion of natural killer cells (NK). IFN-NK cells in secreted substances refer to innate immune cells that can kill cancer cells and virus-infected cells. And IFN-NK cells can regulate the functions of other immune cells and promote tissue growth.

Chemokines are a group of small molecules (mostly 8–10 kD in molecular weight) that are capable of chemotactic cell movement. Chemokines can be classified into four groups according to the number and location of N-terminal cysteine residues: C, CC, CXC, and CX3C. For example, CCL2 chemokine has the ability to chemoattract monocytes, macrophages, and T lymphocytes [14]. It has been shown that anti-CCL2 antibodies blocking the effects of CCL2 such as reducing MDSC, increasing CD4+ and CD8+ T cell infiltration, and promoting IFNy secretion can enhance the tumor immune effect of PD-1 in treating lung cancer in mice [15]. CX3CL1 (FKN, fractalkine) is the only member of the CX3C type chemokine family, which possesses chemotactic functions common to chemokines and mediates the wandering and activation of leukocytes, especially lymphocytes and phagocytes. Li et al. [16] used gene silencing technology to confirm the stable high expression of CX3CL1 in the hepatocellular carcinoma cell line HepG2, which inhibited tumor angiogenesis. In breast cancer studies, CX3CL1 was found to have a possible role in tumor metastasis [17,18], and high CX3CL1 expression correlates with good prognosis in breast cancer [19] and can also be used as one of the indications for immunomodulatory therapy.

In summary, only a few reports have investigated the clinic pathological significance of immune cells in recent years, and the overall expression level and prognostic value of immune cell infiltration, especially in NSCLC patients, require further investigation. Therefore, in this study, we collected data from public databases and constructed statistical scoring models to analyze multiple datasets independently and pooled the analysis results in order to elucidate the prognosis-related molecular mechanisms of NSCLC, which not only helps to further understand the pathogenesis of NSCLC but also provides new ideas and targets for the early diagnosis and treatment of NSCLC.

## 2. Materials and Methods

### 2.1. Preprocessing of Data

The transcriptome and clinical data of NSCLC patients were obtained from the GEO database, using the study cohort GSE1206222 (*n* = 81). The enrolled patients' relevant somatic mutation data were also acquired from the aforesaid database. The original file was used to apply the robust multiarray average (RMA) technique on all of the expression data. Besides, “DeEseq2” and “affy” R packages were used to adjust the background, and log2 conversion was performed for the results of this analysis.

### 2.2. Immune Score, TICS Score, and Pearson Immune Cell Correlation Coefficient Calculation and Immune Cell Infiltration

1. The relative abundance of 28 immune cells of tumor infiltrating in 81 patients with non-small-cell lung cancer was quantified using single-sample gene set enrichment analysis (ssGSEA). The prognosis of 28 immune cells was assessed using Cox risk regression analysis. The TICS value was calculated in terms of the risk value of immune cells and ssGSEA's NES value, and the Z-score was also calculated.
2. We conducted principal component analysis on 81 patients' transcripts. In order to obtain genes related to high infiltration, *T*-test and FDR correction were performed. Then, we acquired upregulated and downregulated genes related to high infiltration and clustered upregulated genes and sample, comparing TICS value and immune score value and calculating Pearson correlation coefficient.

### 2.3. Enrichment Analysis on Function and Path

1. We explored the biological process of these different genes by GO and KEGG functional annotations. In addition, we used the “cluster analysis software” *R* package to implement the characteristic genes based on alternative TICS. Meanwhile, the overall relationship between GO terms and immune cells was acquired by the usage of GSEA.
2. In order to find the relationship between GO terms, the signal pathways related to immune infiltration and the related genes involved were firstly used to construct network by STRING database. Secondly, we mined network modules and analyzed the correlation between genes and the biological processes involved.
3. We examined and projected the survival of patients with non-small-cell lung cancer and drew a survival curve based on immune factor analyses.

## 3. Result 1: Construction of Immune Microenvironment Map of NSCLC

**Figure 1:**
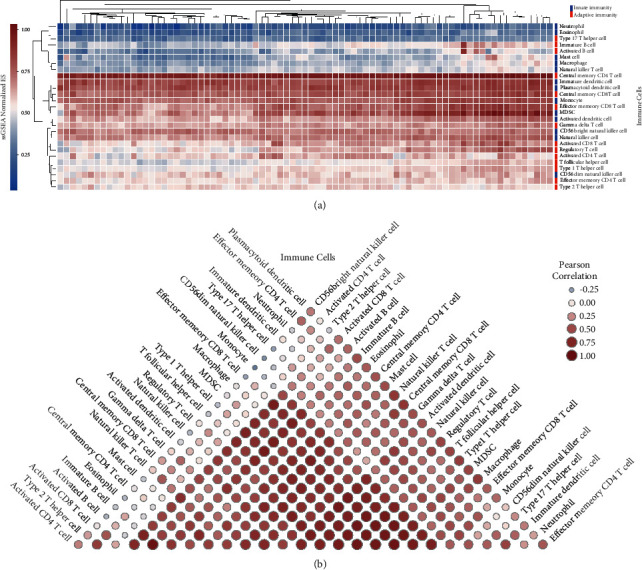
(a) Heat map of immune cell infiltration with the ssGSEA algorithm. (b) Construction of immune cell map based on the Pearson correlation.

One of the most important treatments for non-small-cell lung cancer is immunotherapy (NSCLC). The heterogeneity of immune microenvironment of patients' tissues potentially affects the therapeutic effect. Therefore, the composition of immune cells in tumor tissue must be understood. First, we downloaded the transcriptome data and matched clinical data of 81 patients with NSCLC from GEO database (access number: gse120622). Based on ssGSEA algorithm, we used mRNA expression data of each patient to construct immune cell infiltration maps of NSCLC including 27 types of adaptive and innate immune cells. Central memory CD8 T cell, central memory CD4 T cell, and plasmacytoid dendritic cells are common immune cells in NSCLC tissues, which all exist in 81 patients. In addition, there are some individual specific immune cells, such as immature B cell (Figure 1(a)). We discovered that most immune cells had a high link with activated CD8 T cells, activated B cells, immature B cells, monocytes, effector memory CD8 T cells, and other cells. However, there is no evident link between some immune cells and others, such as the brilliant natural killer cell, activated CD4 T cell, and plasmacytoid dendritic cell (Figure 1(b)). This potentially indicates that there are complex interaction patterns among immune microenvironment of NSCLC patients.

## 4. Result 2: Construction of TICS Scoring Model Based on Immune Microenvironment of NSCLC Patients

**Figure 2:**
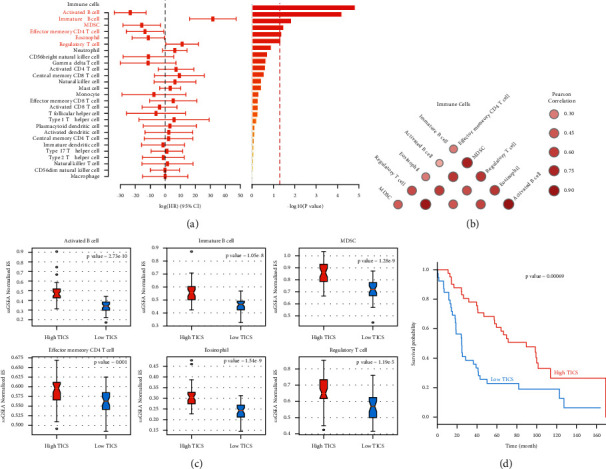


Then, we used Cox regression to analyze and explore the relationship between 27 kinds of infiltrating immune cells and survival time on the basis of the survival data of patients. Meanwhile, we obtained the risk ratio and coefficient of 27 immune cells. The results showed that individual specific immune cells were significantly associated with the survival of NSCLC patients (Figure 2(a)). There was a high correlation between MDSC cells and other five kinds of immune cells (Figure 2(b)). We calculated a TICS score for each patient based on the quantity of these six tumor-infiltrating immune cells (see procedure) and then divided patients into high and low TICS groups based on the median TICS score. In the high TICS group, we discovered that immature B cells, activated B cells, MDSC effector memory CD4 T cells, eosinophils, and regulatory T cells had higher ssGSEA scores (*P* < 0.01) (Figure 2(c)). Various studies have found that the overall survival of many solid tumors, including NSCLC, is positively linked with tumor-infiltrating B cells [8, 20–26]. In addition, tumor-infiltrating B cells and tumor-infiltrating CD4 + T cells created three-level structures of lymphoid that was positively correlated with the survival rate of NSCLC [8, 26]. Furthermore, we analyzed the prognosis of NSCLC patients in accordance with the clinical survival data. We found a substantial difference in prognosis between the two groups, with the high TICS group having a much better prognosis than the low TICS group. This shows that the TICS score constructed can be a reference for clinical prognosis (Figure 2(d)).

## 5. Result 3: Related Genes of TICS Participate in Important Immune Pathways in NSCLC

**Figure 3:**
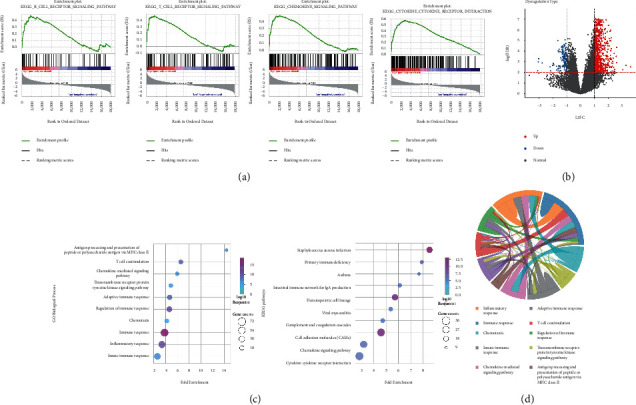


Further study on the biological progress related to TICS will help to analyze the molecular mechanism affecting the prognosis of NSCLC. Firstly, we used mRNA expression data based on TICS group to conduct GSEA in patients with NSCLC. The results showed that the up-regulated genes affecting the prognosis of nonsmall cell lung cancer in the high TICS group were significantly related to the signal pathways of T cells and B cells (Figure 3(a)). This indicates that immune regulation is an important biological process affecting the prognosis of NSCLC, and the upregulated genes in the high TICS group are important molecules involved in its immune regulation. Next, we identified significantly different mRNA (FDR <0.01, FC > 2) between high TICS and low TICS groups based on the mRNA expression data of NSCLC.

Finally, 730 significantly upregulated mRNAs in the high TICS group and 121 significantly downregulated mRNAs in the low TICS group were obtained (Figure 3(b)). In accordance with our results, the upregulated mRNA is more likely to be associated with immune function such as CX3CR1, which mediates the migration and adhesion of leukocytes; CCL21 has no chemotactic effect on B cells, macrophages, or neutrophils. In order to further analyze the function of differential mRNA, we used DAVID tool to enrich it. Gene Ontology (GO) results showed that the upregulated mRNA was enriched in the top 10 biological functions. It mainly includes some function related to immune response. Similarly, the upregulated mRNA was enriched in the top ten pathways of Kyoto Encyclopedia of Genes and Genomes (KEGG). It mainly includes cell adhesion molecules and immune and other related signaling pathways. These results fully show that genes related to immune function can affect the prognosis of NSCLC. In addition, we also explored whether the top 10 biological functions (GO) are related. The result shows that these biological functions are closely related and can share the same gene set, indicating that these biological functions have some similarities.

## 6. Result 4: Immune-Related Genes Are the Key to Distinguish TICS

**Figure 4:**
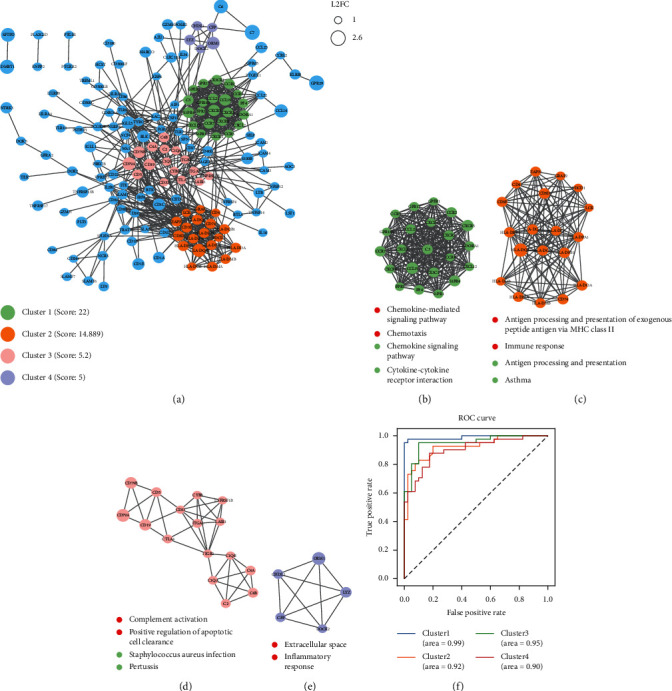


Next, we explored the interactions among the top 10 biologically functional related mRNAs. Firstly, we select high reliability edge (combined score >0.7) from STRING data. Then, 191 mRNAs were matched to the interaction edge. Finally, an undirected network with 164 points and 777 edges is constructed (Figure.  4(a)). Then, we used MCODE plug-in to obtain subnetworks and selected the subnetwork with high reliability for biological function annotation (Figures 4(b)–44(e)). We found that there was a close interaction among mRNA related to signaling pathway mediated by chemokine. At the same time, we explored whether the four subnetworks can effectively distinguish high TICS from low TICS. We find that the four small networks can distinguish high TICS from low TICS by drawing the ROC curve and calculating the area under the curve, indicating that they are potential markers to distinguish TICS (Figure 4(f)). Among them, CCL19 which is as an important member of chemokine-mediated signaling pathway has demonstrated that its expression level in tumor FSC is related to the degree of immune cell infiltration of CD8 + T cells and tumor accumulation (29391257). In conclusion, the level of CCL19 expression is related to tumor size. The lower the expression level, the larger the tumor. Besides, CX3CR1 is also a marker of T cell differentiation (33658501). These chemokines are also significantly associated with the survival of patients with NSCLC. It is reported that the expression levels of CX3CR1, CXCR4, CXCR5, and CCR7 in tumor tissues are significantly increased, which is able to affect the survival time of patients. The 5-year DFS and 5-year OS of patients with positive CCR7 expression are significantly higher (32896997).

## 7. Discussion

Tumor cells, fibroblasts, immune cells, different signal chemicals, and extracellular matrix make up the majority of the tumor microenvironment. Tumor microenvironment significantly affects diagnosis, survival, and clinical treatment sensitivity of tumor. Relevant studies have shown that immune cells of adaptive immune and innate immune system can penetrate into tumor tissue, forming tumor immune microenvironment and affecting tumor progression. Patients' survival following chemotherapy is influenced by the quantity of immune cells in the tumor. In this work, we used non-small-cell lung cancer mRNA expression data to create a microenvironment map of the internal immune system of tumor tissue. Combined with the analysis on immune cells combined with survival data of patients, it was found that there was a significant correlation between the survival of NSCLC patients and immature B cell, activated B cell, MDSC, effector memory CD4 T cell, eosinophil, and regulatory T cell. Next, we constructed the TICS scoring model and graded specific TICS score for patients in terms of prognosis-related immune cell content and its relationship with survival. TICS score can discriminate between patients with non-small-cell lung cancer who have a favourable prognosis and those who have a bad prognosis in terms of survival time.

Furthermore, we identified TICS-related genes by using prognostic markers and mRNA expression profiles. We found that many chemokines were significantly upregulated in the high TICS group. For example, there are CX3CR1, CXCR4, CXCR5, and CCR7 which are commonly associated with the survival of NSCLC. Also, high expression of CX3CR1 and CCL19 can affect the immune differentiation of T cells, such as CD8 + T cells. Previous studies have shown that C3 level in biopsy tissues of NSCLC is certainly correlated (24819254) with infiltrating CD4 +  and CD8 + T lymphocytes. Moreover, the result of functional enrichment showed that TICS-related genes were involved in a variety of immune-related biological progresses and signaling pathways, which indicates that immune-related genes affect the prognosis of NSCLC patients by regulating the immune process.

We established PPIs among immune genes and high-confidence PPI network by using the top ten genes in biological function. PPI network results show that there is a close interaction between these genes, which indicates that immune genes may have complex coordination and interaction by which they can regulate the body's immune response. Furthermore, we explored four important subnetworks in PPI network. By drawing the ROC curve and calculating the area under the curve, we found that these four subnetworks are important molecular markers to distinguish patients from TICS. In conclusion, our study depicts a comprehensive immune map of NSCLC and constructs a TICS scoring model on the basis of the content of immune cells that can effectively distinguish the prognosis time of patients. Differential gene and enrichment analysis explains the prognosis-related molecular mechanism of NSCLC. PPI network and subnetwork mining effectively identified potential markers to distinguish TICS classification. We hope that the prognosis-related immune markers identified in our study will provide guidance for clinical diagnosis, medication, and prognosis prediction of patients.

## Data Availability

The data that support the findings of this study are available from the corresponding author upon reasonable request.
